# Complete Genome Sequence of *Corynebacterium ureicelerivorans* DSM 45051, a Lipophilic and Urea-Splitting Isolate from the Blood Culture of a Septicemia Patient

**DOI:** 10.1128/genomeA.01211-14

**Published:** 2014-11-20

**Authors:** Anna Tippelt, Andreas Albersmeier, Karina Brinkrolf, Christian Rückert, Isabel Fernández-Natal, Francisco Soriano, Andreas Tauch

**Affiliations:** a Institut für Genomforschung und Systembiologie, Centrum für Biotechnologie (CeBiTec), Universität Bielefeld, Bielefeld, Germany; b Department of Clinical Microbiology, Complejo Asistencial Universitario de León (Sacyl), León, Spain; c Institute of Biomedicine (IBIOMED), University of León, León, Spain; d Public Health School of Physiotherapy, Autonomous University of Madrid, Madrid, Spain

## Abstract

*Corynebacterium ureicelerivorans* is an opportunistic pathogen with a lipophilic lifestyle and an exceptionally high urease activity. The genome sequence of the type strain revealed that lipophilism is caused by the lack of a fatty acid synthase gene. The *ureABCEFGD* genes are similar to the urease gene region of *Corynebacterium glucuronolyticum*.

## GENOME ANNOUNCEMENT

*Corynebacterium ureicelerivorans* is a lipophilic coryneform bacterium that is biochemically characterized by the very rapid enzymatic conversion of urea in a standard urease test (1). The taxonomic description of this species is based on a single isolate from the blood culture of a patient suffering from fever and exhibiting symptoms of septicemia (1). *C. ureicelerivorans* is related to *Corynebacterium mucifaciens* and *Corynebacterium pilbarense* by 16S rRNA gene sequence comparison but can be differentiated by DNA-DNA hybridization and biochemical phenotypic methods (1, 2). The *rpoB* gene sequence was determined in six strains of *C. ureicelerivorans*, finding a similarity of 91 to 97% with the type strain of *C. mucifaciens* (3). *C. ureicelerivorans* was recovered from two unspecified clinical samples (4, 5) and from blood cultures and ascitic fluid of patients with digestive disorders (3). Antimicrobial susceptibility assays revealed a common resistance of the latter *C. ureicelerivorans* isolates to macrolide and lincosamide antibiotics (3). To attain genetic knowledge of this disease-associated pathogen, we sequenced the complete genome of the type strain *C. ureicelerivorans* DSM 45051, initially named IMMIB RIV-2301 (1).

*C. ureicelerivorans* DSM 45051 was obtained from the Leibniz Institute DSMZ and was grown at 37°C in brain heart infusion broth-yeast extract medium (6) supplemented with 1% (vol/vol) Tween 80. Genomic DNA was purified with a Genomic-tip 500/G and the Genomic DNA buffer set (Qiagen). A sequencing-ready DNA library was generated with the Nextera DNA sample preparation kit (Illumina) and was sequenced in a 2 × 300 nucleotide paired-end run using the MiSeq reagent kit version 3 (600 cycles) and the MiSeq desktop sequencer (Illumina). This sequencing approach resulted in 3,404,646 paired reads and 818,435,952 detected bases. The paired reads were assembled with the Roche GS De Novo Assembler software (release 2.8), yielding 43 contigs in 36 scaffolds. The scaffolds were ordered by synteny analysis with the r2cat tool (7). The remaining gaps in the genome sequence were closed by PCR assays with the BIOTAQ DNA polymerase (Bioline) and subsequent sequencing of the PCR products was performed on an ABI 3730xl DNA analyzer (Applied Biosystems). The Consed tool (version 26) was used for editing and finishing of the sequence assemblies (8).

The genome of *C. ureicelerivorans* DSM 45051 consists of a circular chromosome with a size of 2,279,990 bp (65.01% mean G+C content) and an extrachromosomal copy of the corynephage ΦCUREI2301I, specified by a size of 48,288 bp and a mean G+C content of 64.56%. The *C. ureicelerivorans* genome lacks a gene encoding a fatty acid synthase, which defines the lipophilism of this species as lipid auxotrophy, just as previously demonstrated for other lipophilic corynebacteria (9–11). A potential macrolide and lincosamide resistance of *C. ureicelerivorans* is encoded by an *erm*(X) gene region showing the best BLASTN hits to a corresponding gene region in *Actinobaculum schaalii* strain 16 from a human urine specimen (12). The urease locus of *C. ureicelerivorans* comprises the *ureABCEFGD* genes revealing the best BLASTX hits to the urease subunits of *Corynebacterium glucuronolyticum*, isolated from the genital tract of humans (13, 14).

### Nucleotide sequence accession numbers.

This genome project has been deposited in the GenBank database under the accession numbers CP009215 (chromosome) and CP009216 (ΦCUREI2301I).
